# Psilocybin’s effect on human brain synaptic plasticity

**DOI:** 10.1038/s41398-026-04285-y

**Published:** 2026-07-15

**Authors:** Annette Johansen, Pontus Plavén-Sigray, Martin K. Madsen, Anna Søndergaard, Catharina Messel, Maria Geisler, Arafat Nasser, Drummond E-W. McCulloch, Vincent Beliveau, Alexandra Vassilieva, Anton Lund, Szabolcs Lehel, Brice Ozenne, Dea S. Stenbæk, Patrick M. Fisher, Claus Svarer, Gitte M. Knudsen

**Affiliations:** 1 Neurobiology Research Unit, Rigshospitalet, Copenhagen University Hospital, Copenhagen, Denmark; 2 Department of Neurology, Rigshospitalet, Copenhagen University Hospital, Copenhagen, Denmark; 3 Centre for Psychiatry Research, Department of Clinical Neuroscience, Karolinska Institutet & Stockholm Health Care Services, Region Stockholm, Solna, Sweden; 4 Department of Psychiatry Odense-Svendborg Hospital, Svendborg, Denmark; 5 Department of Clinical Medicine, Faculty of Health and Medical Sciences, University of Copenhagen, Copenhagen, Denmark; 6 Institute for Human Genetics, Medical University of Innsbruck, Innsbruck, Austria; 7 Department of Neuroanesthesiology, Rigshospitalet, Copenhagen University Hospital, Copenhagen, Denmark; 8 PET & Cyclotron Unit, Rigshospitalet, Copenhagen University Hospital, Copenhagen, Denmark; 9 Department of Public Health, Section of Biostatistics, University of Copenhagen, Copenhagen, Denmark; 10 Department of Psychology, Faculty of Social Sciences, University of Copenhagen, Copenhagen, Denmark; 11 Department of Drug Design and Pharmacology, University of Copenhagen, Copenhagen, Denmark

**Keywords:** Biomarkers, Molecular neuroscience

## Abstract

Psychedelics such as psilocybin have been linked to enhanced neuroplasticity and symptom relief in affective disorders, but the neurobiological mechanisms and impact of environmental context remain unclear. Here, we tested whether a single dose of psilocybin (0.3 mg/kg) alters synaptic density in healthy individuals and whether setting-dependent subjective experience shapes this effect. Fifteen healthy participants had a psilocybin-induced psychedelic experience either inside an MRI scanner or in a therapeutic-like room. We assessed synaptic density changes by measuring the Synaptic Vesicle glycoprotein 2A in the frontal cortex and hippocampus with [¹¹C]UCB-J PET at baseline and one-week post-dose, and assessed subjective experiences immediately afterwards and at three months. Overall, we found no statistically significant increase in SV2A density. However, participants treated in the therapeutic-like setting exhibited more intense mystical-type experiences, longer-lasting psychological benefits, and greater increases in synaptic density than those dosed in the MRI scanner. These findings indicate that psilocybin’s neuroplastic effects are modulated by environmental context, with important implications for psychedelic-assisted therapies. **ClinicalTrial**: NCT03289949.

## Introduction

Classic psychedelics such as psilocybin and lysergic acid diethylamide (LSD) are emerging as potential therapeutics for treating a range of neuropsychiatric disorders [1]. The compounds stimulate the serotonin 2A receptor (5-HT_2A_R), which acutely induces a dose-dependent altered state of consciousness including perceptual, emotional, and cognitive changes [2, 3]. Intake of only one to two doses of psilocybin is associated with long-lasting positive emotional effects in healthy individuals [4, 5] and symptom improvement in patients with major depressive disorder (MDD) [6–8], anxiety [9], addiction [10, 11] and cluster headache [12].

A compelling hypothesis for the rapid therapeutic action of psychedelics is their ability to induce neuroplasticity [13, 14]. The psychological plasticity which manifests through increased cognitive flexibility and psychological insight [15–17] has been suggested to be linked to functional and microstructural changes in the brain, a view that has been bolstered by a growing body of preclinical studies [18–21].

The neuroplastic effects of psychedelics are coupled to activation of the 5-HT_2A_R; when cultured neurons are stimulated with 5-HT_2A_R agonists, structural and functional changes such as dendritic sprouting and arborization and upregulation of presynaptic markers emerge [21]. This happens as early as six hours after 5-HT_2A_R stimulation, with persisting effects detected up to seven days later [22–24]. One week after awake pigs are given a single dose of i.v. psilocybin, they display higher density of the presynaptic marker, Synaptic Vesicle glycoprotein 2A (SV2A), in the hippocampus and frontal cortex, as compared to saline-treated animals [25]. SV2A can be quantified in vivo in the human brain using positron emission tomography (PET) with the tracer [^11^C]UCB-J [26]. In the human cerebral cortex, SV2A is highly expressed with a regional distribution reflecting higher-order human brain functions [27]. Results from our previous study suggest that 3–5 weeks of intake of the selective serotonin reuptake inhibitor, escitalopram, is associated with higher SV2A levels in a time-dependent manner, supporting that the *delay* in therapeutic action is associated with an increased synaptic density, putatively reflecting structural plasticity [28]. In contrast, the positive psychological effects of psilocybin occur *immediately* following the dosing session and persist long after the drug has been cleared from the body. Positive emotional experiences during the session, as captured by the *Mystical Experience Questionnaire* [29] and the *Oceanic Boundlessness* facet of the *Altered State of Consciousness* Q*uestionnaire* [30] have been linked to better therapeutic outcomes in patients [9, 31–33] and lasting positive effects in healthy individuals [34]. A central question is how aspects pertaining to the psychedelic experience relate to neuroplasticity and lasting positive effects. In a single-arm, open-label study, we investigated whether a single psychedelic dose of psilocybin increases cerebral SV2A levels, as measured with [¹¹C]UCB-J PET, in healthy individuals, by estimating SV2A density in the frontal cortex and the hippocampus before and one week after the session. We further explore how changes in SV2A density relate to the settings of the psilocybin session and subjective effects during the psychedelic experience.

## Methods

### Participants

Fifteen healthy volunteers were recruited from a local database of individuals who had expressed interest in participating in studies involving psilocybin. Following informed consent, participants underwent extensive screening procedures including somatic and psychiatric medical history, clinical examination, standard blood biochemistry, and an electrocardiogram to exclude the presence of prior or current major somatic or psychiatric disorder. Full list of exclusion criteria can be found in the supplemental materials.

The study was approved by the Danish Medicines Agency (EudraCT ID: 2016-004000-61, amendments: 2020061833, 2021041519) and by the ethics committee for the Capital Region of Denmark (ID: H-16028698, with amendments). The study was preregistered at ClinicalTrials.gov (NCT03289949, subproject 2b). Alle study elements were performed in accordance with the relevant guidelines and regulations.

### Study design and psilocybin intervention

The study was designed as an open label, group-sequential single arm intervention study. A power-analysis was performed, suggesting that n = 15 participants would be able to detect a 5% increase in [^11^C]UCB-J *V*_T_ with a statistical power of 0.8, using a one-sided, paired t-test at significance level 0.05. A single interim analysis was planned at 80% of the recruitment (n = 12 individuals with complete data), with the possibility to stop the study for efficacy, or futility, or continue recruitment until n = 15 individuals with complete data. Participants underwent baseline Magnetic Resonance Imaging (MRI) and [^11^C]UCB-J PET scans on the same day (in no specific order), approximately one week before undergoing an intervention session with peroral psilocybin (0.3 mg/kg). This dose was selected based on our previous human PET studies, where this dose was found to be safe and well-tolerated while reliably producing intense psychedelic experiences [3]. The time point of one week was selected specifically to investigate persistent effects, aligned with our lab’s previous preclinical study suggesting that this time point is associated with an increase in SV2A [25].

The psilocybin sessions took place in either an MRI scanner with a series of functional MRI acquisitions including active (*emotional faces*) and passive (e.g., resting-state fMRI, music listening) paradigms (participants 1–5; 4M/1F), or in a designated intervention room while listening to a curated music playlist constructed to facilitate the different phases of the psychedelic experience [35] (participants 6–15; 6M/4F). One week after the psilocybin session, participants returned for follow-up [^11^C]UCB-J PET and MRI scans.

We used a structured psychological support model consisting of preparation, support during dosing, and an integration session, delivered by two facilitators (psychologists or supervised MSc students). No formal psychotherapy was provided.

Preparation focused on establishing rapport, discussing expectations and motivations, and providing information about study logistics, dosing-day procedures, expected psilocybin effects, and strategies for managing challenging experiences. Boundaries regarding supportive touch and session ground rules were established. On the intervention day, participants received a 40–60-min pre-dosing session in either the intervention room or a preparation room adjacent to the MRI scanner. Facilitators remained present throughout the psilocybin session, offering interpersonal support, affect regulation, emotional validation, and practical assistance (e.g., food, beverages, bathroom visits). Facilitators encouraged participants to direct attention inward and be guided by the music; verbal communication during the peak of the drug effects was sparse. For participants undergoing dosing in the MRI scanner, one facilitator remained in the scanner room throughout imaging (typically providing reassuring physical contact by placing a hand on the participant’s leg) while both facilitators were present in the adjacent room during breaks between scans, offering psychological support and the option of music listening. After subjective drug effects subsided, facilitators reviewed immediate impressions, addressed self-care needs, and ensured participants were ready to leave in collaboration with medical staff. The following day, all participants completed an integration session to reflect on the experience, discuss early post-session changes, and support meaning-making and incorporation into daily life.

### Subjective effects questionnaires

Psychological questionnaires evaluating the acute effects of psilocybin were completed on the day of the psilocybin session, after psychedelic effects had subsided, and included the Altered State of Consciousness Questionnaire (5D-ASC, scale 0–100) [30] and the Revised Mystical Experience Questionnaire-30 (MEQ30, scale 0–5) [29]. From the 5D-ASC questionnaire, 11 subscales were created from the dimensions Oceanic Boundlessness (OBN) which assesses “mystical-type experiences” (comprising the subscales *Experience of unity, Blissful state, Spiritual experience, Insightfulness, and Disembodiment*); Dread of Ego-Dissolution (DED) which is concerned with negative experiences and anxiety (comprising the subscales *Anxiety* and *Impaired control and cognition*); and Visionary Restructuralization (VRS) assessing effects on sensory perception (comprising the subscales *Complex imagery, Elementary imagery, Audio-visual synesthesia, Changed meanings of percepts*). The MEQ30 shares features with the OBN dimension of the ASC and contains the subscales *Mystical*, *Positive mood*, *Transcendence of time and space*, and *Ineffability*. Persisting effects were evaluated three months after the psilocybin session using the Persisting Effects Questionnaire (PEQ) [36], which assesses positive and negative effects on life, self, mood, behavior, social effects, and spirituality attributed to the psychedelic experience.

### Imaging acquisition and processing

MRI data were acquired on a Siemens Magnetom Prisma 3T scanner using a 32-channel head coil (Siemens AG, Erlangen, Germany). PET data were acquired over 120 min with the high-resolution research tomograph (HRRT) PET scanner (CTI/Siemens, Knoxville, TN, USA) with collection of arterial blood samples to establish a metabolite-corrected arterial input function. Details on MRI and PET parameters and processing are provided in the supplementary file.

### Kinetic modeling of PET and blood data

Kinetic modeling of [^11^C]UCB-J PET data was performed in R (v. 4.2.2, R Foundation, Vienna, Austria) using the *kinfitr* package v. 0.6 [37]. Regional time-activity curves from the hippocampus and frontal cortex (volume-weighted average of orbitofrontal cortex, medial and inferior frontal gyrus, superior frontal gyrus, anterior cingulate gyrus, precentral gyrus) and the corresponding metabolite-corrected arterial input function were fitted to the one-tissue compartment model (1TCM) to estimate the total volume of distribution (*V*_T_). As an alternative outcome parameter, the time-activity curves were fitted using the simplified reference tissue model 2 (SRTM2) to estimate the non-displaceable binding potential (*BP*_ND_) using the white matter region centrum semiovale as the reference region, and the median k2 from 1TC modeling of centrum semiovale as a global k2’ as determined within the sample.

### Statistical analyses

For the primary analysis, mean changes in [^11^C]UCB-J *V*_T_ and *BP*_ND_ between baseline and one week follow-up were assessed using one-tailed paired t-tests for the frontal cortex and the hippocampus to test the hypothesis that SV2A binding increase after psilocybin intervention in accordance with the pre-registration protocol (aspredicted.org ID: 93092, see supplementary material). We also pre-registered an interim test at n = 12/15 (information fraction = 0.8) with an adjusted significance level of 0.028, allowing us to stop for efficacy or futility (conditional power <50%). Tests with one-sided *p*-values are referred to as *p*_one-sided_.

**Fig. 1:**
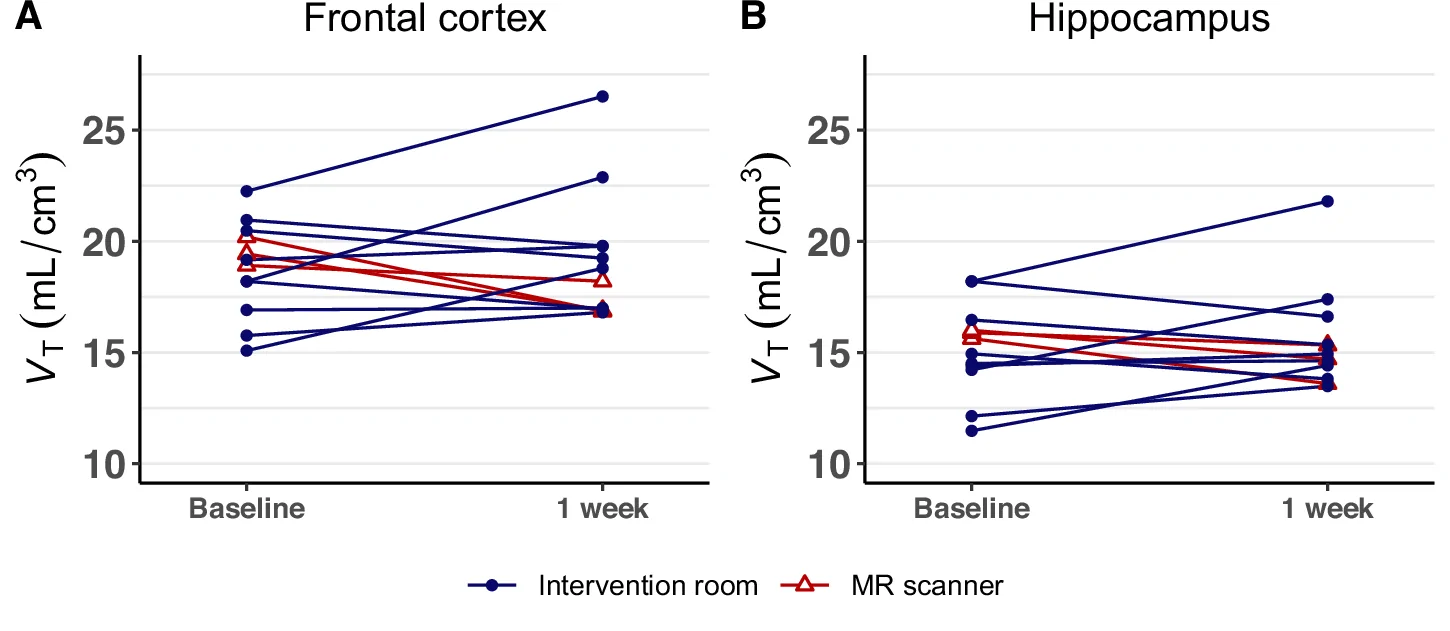
Effects of psilocybin on presynaptic density. SV2A binding quantified as [^11^C]UCB-J *V*_T_ for the frontal cortex **A** and the hippocampus **B** at baseline and 1-week after the psilocybin intervention.

In exploratory analyses, we investigated (1) whether the self-reported MEQ30 and 5D-ASC scores rated immediately after the intervention and PEQ rated three months after the intervention differed between session environments, (2) whether change in SV2A binding differed between session environments, and (3) whether change in SV2A binding was associated with the subjective intensity of mystical-type experiences and persisting effects. For these analyses, we applied a linear latent variable model (LVM) (see Fig. 1 for graphical representation) [38] with a latent variable, *Mysticality*, to capture the shared correlation between the ASC subscales *Experience of Unity, Spiritual Experience, Blissful State, Insightfulness*, and *Disembodiment* combined with the MEQ sub-scales (rescaled to 0–100) *Mystical, Positive Mood, Transcendence of Time and Space*, and *Ineffability*. For the persisting effects, we applied latent variable models with the latent variables *Positive Persisting Effect* and *Negative Persisting Effects*, to capture the shared correlation between the positive and negative PEQ subscales, respectively (see Fig. S1 for graphical representation). A separate parameter was used in each model to estimate the mean latent variable scores in the MRI scanner and intervention room. Linear latent variable models estimate how multiple observed measures, such as questionnaire subscales, reflect a common underlying construct, enabling quantification of associations between underlying psychological factors and biological outcomes while controlling for measurement noise. Because standard LVM tests exhibit inflated type 1 error in small samples [39], permutation tests with 10,000 permutations were performed to compute the *p*-values (referred to as *p*_perm_).

For the [^11^C]UCB-J *V*_T_s we compared a random-intercept linear mixed-effects model including the session environment as an interaction term to a model without the session environment using a likelihood ratio test, where participant ID was modeled as a random effect and time (baseline vs. follow-up), and session environment as fixed effects. The frontal cortex and the hippocampus were modelled separately. We next examined the association between the mysticality latent variable scores and the [^11^C]UCB-J *V*_T_s.

## Results

### Sample description and psychedelic effects

Participant demographics and summary statistics of [^11^C]UCB-J PET scan parameters are presented in Table 1. Injected dose, weight-adjusted injected mass, and free fraction of [^11^C]UCB-J were comparable between baseline and the one-week follow-up. Alcohol consumption was low to moderate overall, with a mean weekly intake of 6 units (range 0–15). Lifetime substance use was common and primarily limited to cannabis, which was reported by 10/15 participants, followed by psilocybin-containing mushrooms reported by 4/15. Cocaine use was reported by 2/15 participants, and MDMA use by 1/15.

**Table 1:** Subject demographics and PET scan-related parameters.

	Complete PET data^a^ N = 12	Full sample N = 15
**Age (years)**	31.5 [26.6, 53.7]	32.5 [26.6, 60.3]
**Female/male**	3/9	5/10
**Intervention room (n, %)**	9 (75%)	10 (66%)
**Complete PET scans, baseline/rescan**	12/12	13/12
**Injected radioactivity dose (MBq)**		
**Baseline**	415 [266, 479]	413 [266, 481]
**1-week follow-up**	399 [89, 466]	388 [89, 466]
**Injected mass (ng/kg)**		
**Baseline**	5.7 [2.7, 10.8]	5.7 [1.5, 10.8]
**1-week follow-up**	3.9 [2.2, 10.6]	3.6 [2.2, 10.6]
***f***_**p**_		
**Baseline**	0.35 [0.22, 0.45]	0.33 [0.22, 0.45]
**1-week follow-up**	0.33 [0.21, 0.44]	0.33 [0.21, 0.44]

^a^Complete PET data includes subjects with complete PET and arterial blood data at both baseline and follow-up. Values are presented as median [range]. *f*_P_ : free fraction of [^11^C]UCB-J in plasma.

Psilocybin interventions were generally well-tolerated in both session environments. One participant in the intervention room briefly experienced anxiety as the drug’s effects emerged but responded well to psychological support. No other participants experienced adverse effects related to the intervention.

### Effects of psilocybin on cerebral SV2A levels

The interim analysis, based on 12 participants with complete arterial blood data, showed no statistically significant increase in *V*_T_ following the psilocybin intervention. The conditional power analysis indicated 24% probability of reaching significance at study completion with the originally hypothesized effect size, and 2% probability with the observed effect size. As this fell below our pre-registered futility threshold of 50% conditional power, the study was terminated for futility.

For these 12 participants, the mean difference (SD) in *V*_T_ was 0.3 (2.6) mL/cm^3^ (*p*_one-sided_ = 0.29) corresponding to 2.3% in the frontal cortex, and 0.3 (2.0) mL/cm^3^ (*p*_one-sided_ = 0.33) corresponding to 3.0% in the hippocampus (Fig. 1). *BP*_ND_s calculated for all 15 participants showed a mean difference (SD) of −0.01 (0.35) (*p*_one-sided_ = 0.54) corresponding to 0.03% in the frontal cortex, and 0.01 (0.28) (*p*_one-sided_ = 0.44) corresponding to 1.01% in the hippocampus (Fig. S2).

### Exploratory analyses

**Fig. 2:**
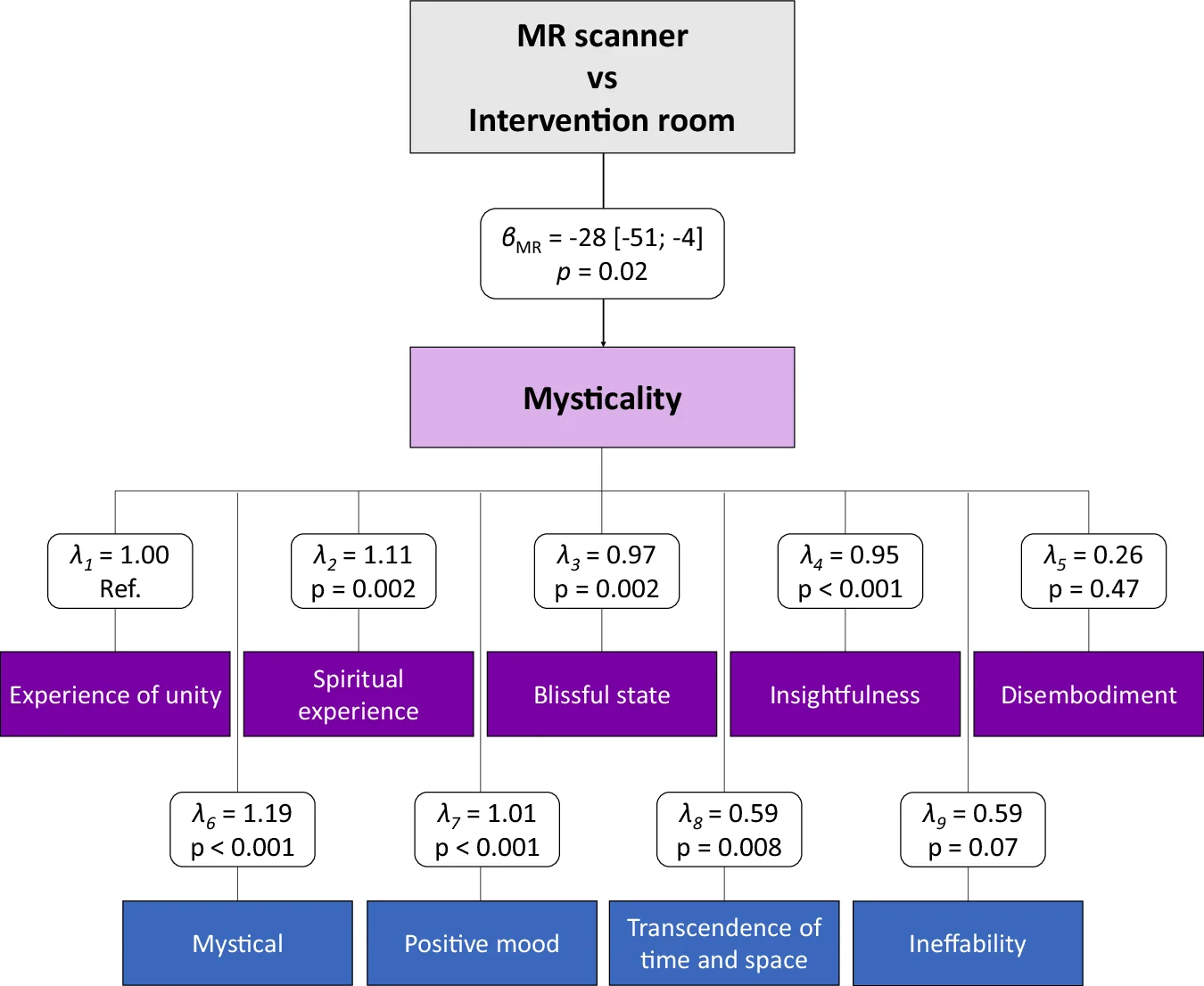
Latent variable model comprising the ASC-OBN subscales (purple boxes) and MEQ subscales (blue boxes) to capture the latent variable *mysticality* in subjective psychedelic experiences. Undergoing the psilocybin session in the MR scanner was associated with less intense mystical-type experiences.

We examined whether the session environments were associated with differences in participants’ subjective mystical-type experiences during the psilocybin session and their persisting effects three months after the session. Using an LVM (Fig. 2), we found that participants who underwent the psilocybin session in the MR environment reported significantly lower intensity of mysticality (*β*_MR_ = −28, *p*_perm_ = 0.02). The ASC-OBN subscales showed similar contribution to the latent variable with loadings close to 1 and highly significant *p*-values, except for *Disembodiment*. The MEQ subscales *Transcendence of time and space* and *Ineffability* also showed low loading weights (0.59 for both), whereas the other loadings were close to 1. The distribution of individual subscale raw scores is shown in Figs. S3–4.

Similarly, the participants who underwent the psilocybin session in the MR environment reported significantly lower positive persisting effects (*β*_MR_ = −33, *p*_perm._ = 0.01, Fig. S1), while negative persisting effects did not differ significantly (*β*_MR_ = +99% (log-transformed), *p*_perm._ = 0.29, Fig. S1).

Given the significant differences in both intensity of mystical-type experiences and positive persisting effects between the participants in the intervention room and those in the MR scanner, we examined whether SV2A binding changes (Δ*V*_T_s) differed between the subgroups. A likelihood ratio test comparing a model with and without the session environment variable as an interaction term showed an effect of the session environment on Δ*V*_T_s for the frontal cortex (*p* = 0.03) and the hippocampus (*p* = 0.09). The difference in Δ*V*_T_ between the two session environments was 3.0 mL/cm^3^ (95% CI [0.4;5.5]) in the frontal cortex and 1.9 mL/cm^3^ (95% CI [−0.3;3.9) in the hippocampus. For participants in the intervention room, *V*_T_s increased from baseline to 1-week follow-up by 1.1 mL/cm^3^ (95% CI [−0.5;2.7]) in the frontal cortex and 0.8 mL/cm^3^ (95% CI [−0.5;2.1]) in the hippocampus, corresponding to a 5–6% increase.

Next, we investigated whether changes in SV2A binding were associated with mystical-type experiences during the session or persisting effects three months later. A linear mixed-effects model estimated a nominal increase in SV2A binding of 0.5 mL/cm^3^ per 10 units increase in mysticality scores (from the LVM shown in Fig. 1) for the frontal cortex (*p* = 0.16) and 0.3 mL/cm^3^ for the hippocampus (*p* = 0.28).

We also examined whether there was an association between the latent variable *positive persisting effects* (Fig. S1) and SV2A binding changes: *β* = +2.6 points per mL/cm^3^ for both the frontal cortex and the hippocampus (*p* > 0.37). The association with *negative effects* (log-transformed, Fig. S1) was −21% per mL/cm³ (*p* = 0.05).

## Discussion

This study is the first to investigate whether a single psychoactive dose of psilocybin induces synaptic plasticity in the healthy human brain. While the overall increase in cerebral SV2A density was not statistically significant one week after the psilocybin session, the session setting emerged as a key determinant of both neurobiological and psychological outcomes. Participants who underwent the session in a designated calm intervention room with curated music showed a 5–6% increase in SV2A binding in the frontal cortex and hippocampus, aligning with the study’s targeted effect size, whereas those scanned during the session did not show this increase. Given the small sample and limited statistical power, particularly in the MR subgroup, observed differences in SV2A changes should be considered preliminary and hypothesis-generating rather than conclusive.

The setting also affected subjective experiences; participants in the MR scanner reported lower scores on mystical-type experiences and had lower positive persisting effects three months later. Moreover, our exploratory analyses found a numerical increase in SV2A binding with higher mysticality score. This observation should be further investigated as a predefined hypothesis in future studies, including both larger sample sizes and in patients with, e.g., major depressive disorder. Together, these results highlight that the psychological context – encompassing the physical environment, emotional support, and music – can meaningfully shape both the subjective and biological effects of psilocybin. Our findings suggest that in response to psilocybin, synaptic plasticity may be influenced by supporting psychological effects of the setting and the emotional valence of the psychedelic experience.

Mystical-type experiences during psilocybin interventions have been linked to long-term positive effects, including enhanced well-being in healthy individuals [34, 40], reduction in anxiety and depressive symptom in patient with major depressive disorder [32, 33] and terminal cancer [9, 31]. While these effects could result from differences in psilocybin dosage and subjective drug intensity, prior studies suggest otherwise. Roseman et al. did not find similar effects of setting on sensory perceptual experiences, and Griffiths et al. reported that the effect of setting on mystical experiences remained significant even after adjusting for drug intensity. Additionally, a recent study in 233 patients with depression found that the association between symptom reduction and *Oceanic Boundlessness* scores remained consistent across three different psilocybin doses (1, 10, and 25 mg) [33]. Mediation analyses by both Ross et al. and Griffiths et al. also support that therapeutic effects are partially driven by mystical-type experiences [9, 31].

Thus, the positive effects of psilocybin in both healthy individuals and psychiatric patients may be linked to neurobiological modulations of synaptic connections that depend on the acute subjective effects. The emotional valence of these experiences appears particularly important, not only when comparing mystical-type experiences (e.g., ASC-OBN, MEQ30) to sensory-perceptual changes, but also when looking into the subscales within these facets. Our latent variable model (Fig. 1) showed that subscales with less emotional valence (*Disembodiment*, *Transcendence of Time and Space*, *Ineffability*) had the lowest weighting. This is consistent with *Disembodiment* being the only OBN subscales that did not differ between responders and non-responders in psilocybin-assisted depression treatment [32]. Similarly, McCulloch et al. found that while the MEQ30 predicted positive effects at three months, the subscales *Transcendence of Time and Space* and *Ineffability* were not statistically significant [34].

Collectively, these findings suggest that psilocybin’s potential to induce enduring psychological and neurobiological changes depends critically on the quality of the acute emotional experience and the therapeutic setting in which it occurs. A supportive environment aims to facilitate positive affect, a sense of safety, and openness – conditions that could promote adaptive network reorganization and consolidation of new synaptic connections. Conversely, stressful or distracting settings, such as the MRI environment, may attenuate these processes by limiting the emergence of emotionally meaningful or integrative experiences.

Some important aspects of the present study should be considered. We conducted the study in healthy individuals who may exhibit less pronounced synaptic plasticity-promoting effects due to homeostatic plasticity mechanisms [41]. In contrast, patients with major depressive disorder show lower SV2A binding, with deficits correlating with symptom severity [42]. Thus, psilocybin may induce stronger synaptic upregulation in clinical populations, potentially restoring lost synapses, while exerting smaller effects in healthy individuals.

The timing of the follow-up PET scan is another important consideration. Preclinical studies have shown that psychedelics can induce neuroplasticity within 24 h [21]. However, a human PET study of ketamine found no SV2A changes 24 h post-administration [43]. Our choice of a one-week follow-up scan was informed by data from pig brain studies finding that synaptic upregulation is higher one week than one day after psilocybin [25]. It is likely that the observed increase in SV2A in humans reflects a transient state of synaptic plasticity and early remodeling, which subsequently stabilizes into a new network configuration shaped by ongoing neural activity. The long-term expression of such changes may depend on the interplay between plasticity and homeostatic mechanisms [43], as well as on psychological factors and life circumstances that can either sustain or diminish these adaptations. Few other studies have reported in vivo SV2A changes following pharmacological interventions in humans. We recently reported that intake of the selective serotonin reuptake inhibitor escitalopram was associated with a time-dependent effect on SV2A, suggesting a gradual enhancement of synaptic density [28]. Similarly, recent cocaine use has shown an association with higher SV2A density [44], whereas cannabis use is not [45]. As such, different classes of psychoactive drugs may have distinct and time-varying effects on synaptic plasticity, with stimulants and psychedelics promoting synaptic upregulation or synaptic vesicle capacity through different mechanisms.

Some methodological limitations of the study should be noted. Our paired PET scan design enables within-subject comparisons and was powered based on a same-day test-retest study. However, two other SV2A neuroimaging studies with healthy individuals have reported lower SV2A in the second scan session; both when re-scanning the same day [46] or four weeks later [47]. Both these studies found lower *V*_T_ values at the second scan, in the range of −1 to −8%, and for *BP*_ND_ in the range of −5 to −14%. The underlying mechanism of this bias remains unclear, but in the absence of a placebo arm, we may have underestimated the effect of psilocybin to increase SV2A. In future studies examining synaptic density and treatment response in clinical populations, inclusion of a placebo group will be essential to control for test-retest effects, better isolate drug-specific changes, and allow more definitive conclusions regarding treatment-related synaptic plasticity. Second, in our group sequential design, the main analysis used a stricter significance threshold (p = 0.028) to maintain 5% type I error rate. We did not pre-specify thresholds for exploratory analyses; adjustment depends on the correlation with the primary outcome – 0.05 may be appropriate for independent outcomes, while 0.028 applies to highly correlated ones. However, since the trial stopped for futility rather than efficacy, using an unadjusted threshold is less concerning. Finally, we did not account for uncertainty in the LVM-weighted scores, which may underestimate *p*-values.

## Conclusion

Our study represents an important step in understanding the persisting effects of psilocybin on the human brain. We show that the psychedelic experience is shaped by the session environment and present the first evidence that synaptic plasticity may be enhanced when the intervention takes place in a setting that facilitates mystical-type experiences.

## Supplementary information

Supplementary file

As predicted

## Data Availability

The data that support the findings of this study are available from the corresponding author upon reasonable request. Individual participant data are not publicly available because of ethical and privacy restrictions.
