# Supplementary material for: Psilocybin’s effect on human brain synaptic plasticity
Source: Transl Psychiatry. 2026 Jul 15;16:544. doi: 10.1038/s41398-026-04285-y (PMC13650139; doi:10.1038/s41398-026-04285-y)
Supplement: Supplementary file 1 [file 41398_2026_4285_MOESM2_ESM.pdf]

Does psilocybin induce synaptic plasticity in the human brain? (#93092)

Author(s)
This pre-registration is currently anonymous to enable blind peer-review.
It has 5 authors.

Pre-registered on: 2022/04/04 - 08:23 AM (PT)

1) Have any data been collected for this study already?
It's complicated. We have already collected some data but explain in Question 8 why readers may consider this a valid pre-registration nevertheless.

2) What's the main question being asked or hypothesis being tested in this study?
Does Synaptic Vesicle glycoprotein 2A as measured with [11C]UCB-J PET increase 1 week after psilocybin intervention session in healthy volunteers?

3) Describe the key dependent variable(s) specifying how they will be measured.
[11C]UCB-J binding in the brain is the key dependent variable. Specifically, they key dependent variable will be 1) 1TCM based VT values derived using an arterial input function and 2) SRTM2 BPND values estimated using cerebral white matter (i.e., centrum semiovale) as the reference region. The hippocampus and frontal cortex (FC) will be primary analyses and other brain regions exploratory).

4) How many and which conditions will participants be assigned to?
All participants will be assigned to one condition and undergo a [11C]UCB-J PET scan before and after an intervention session wherein they will receive an oral dose of psilocybin with psychological support.

5) Specify exactly which analyses you will conduct to examine the main question/hypothesis.
The confirmatory analyses will be :
1) a one-sided paired t-test of the difference between pre and post intervention [11C]UCB-J 1TCM VT in FC and hippocampus respectively, to test the hypothesis that psilocybin increases SV2A binding, as measured with [11C]UCB-J PET. As VT in FC and hippocampus is highly correlated, no correction for multiple testing will be performed. See point 7 for the chosen significance level.
2) a one-sided paired t-test of the difference between pre and post psilocybin intervention [11C]UCB-J SRTM 2 BPND in FC and hippocampus respectively, to test the hypothesis that psilocybin increases SV2A binding, as measured with [11C]UCB-J PET. Since BPND in FC and hippocampus is highly correlated, no correction for multiple testing will be performed. See point 7 for the chosen significance level.
If there is a significant difference in free fraction between pre- and post-psilocybin, then it will be accounted for in the modelling of [11C]UCB-J VT values.

6) Describe exactly how outliers will be defined and handled, and your precise rule(s) for excluding observations.
Outlier detection will be performed in a blinded fashion (pre or post scan status unkown) on blood-data curves and ROI-TAC level. If blood time-activity curves show a large visual divergence from the group average and dispersion, radiochemistry will be re-assessed and re-quality controlled. If critical blood data measurements fail (e.g., impossible to establish a metabolite-curve), VT values will not be estimated for such scans.

7) How many observations will be collected or what will determine sample size? No need to justify decision, but be precise about exactly how the number will be determined.
A power-analysis was performed before the start of data collection (see point 8), suggesting that N = 15 participants would be able to detect a 5% increase in [11C]UCB-J VT with a statistical power of 0.8, using a one-sided, paired t-test. An intermittent analysis will be performed at N = 12 participants (information fraction = 0.8) scanned pre and post intervention using a one-sided O'Brian-Flemming boundary corresponding to significance level of 0.028 for test 1 and 2 (see point 5). If this analysis is not statistically significant, a final analysis will be carried out at N = 15 participants (information fraction = 1) with a one-sided O'Brian-Flemming boundary corresponding to a significance level of 0.042 in order to maintain a type-I α = 0.05 for test 1 and test 2 (see point 5), respectively.
In addition, we will perform a test for futility at N = 12 (information fraction = 0.8) to decide if we will stop the recruitment; if the conditional power is so low that the probability of rejecting the null-hypothesis at N = 15 (information fraction = 1) is <50%, for test 1 and 2 (see point 5), respectively.

8) Anything else you would like to pre-register? (e.g., secondary analyses, variables collected for exploratory purposes, unusual analyses planned?)
The imaging data (PET and MR scans) are currently being collected, but no statistical analyses or data visualizations have been performed yet. Importantly, the preprocessing, image analysis and pharmacokinetic modelling is performed automatically and blinded for condition (pre-post intervention, see below). Hence, nothing pre-registered in this document has yet been examined, and to the best of our knowledge, no subjective procedure is employed in the research project that could affect the statistical results of a pre-post intervention difference in [11C]UCB-J binding towards a biased finding or conclusion.
In addition to the confirmatory analyses mentioned above, we will also include some post-hoc analyses that will be clearly reported as 'exploratory' or 'post-hoc'.
These analysis will, e.g., aim at addressing the hypothesis that psilocybin sessions will lead to an increase in [11C]UCB-J VTs in individuals who meet certain thresholds for SDI, MEQ, MAAS or plasma psilocin concentrations and we may also want to test if there is a positive linear association between a psilocybin-induced increase in [11C]UCB-J VTs and MEQ or MAAS. Other regions than frontal cortex and the hippocampus may be investigated as well.
