# Supplementary material for: Psilocybin’s effect on human brain synaptic plasticity
Source: Transl Psychiatry. 2026 Jul 15;16:544. doi: 10.1038/s41398-026-04285-y (PMC13650139; doi:10.1038/s41398-026-04285-y)
Supplement: Supplementary file 2 [file 41398_2026_4285_MOESM1_ESM.docx]

Supplementary material

# Supplementary methods

List of exclusion criteria

1) Current or previous primary psychiatric illness (DSM IV Axis I or WHO ICD-10 diagnostic classification).

2) Current or previous primary psychiatric illness (DSM IV Axis I or WHO ICD-10 diagnostic classification) in immediate relatives (parents and siblings).

3) Present or previous neurological illness, somatic severe illness, or use of medication that may be assumed to influence the test results.

4) Non-fluent in Danish or pronounced visual or hearing impairment.

5) Information about current or previous learning difficulties (including dyslexia).

6) Pregnancy on the day of the scan, which is ensured by performing a pregnancy test prior to the experiment on each day of the experiment.

7) Breastfeeding.

8) Contraindications for MRI scanning (pacemaker, claustrophobia, etc.).

9) Alcohol or drug abuse.

10) Allergy to ingredients in the drugs used.

11) Participation in experiments involving radioactivity (>10 mSv) within the last year or significant occupational exposure to radioactivity.

12) Abnormal ECG (ECG indicating current or previous heart disease or predisposition to heart disease, especially QT prolongation) or use of QT-prolonging medication

13) Previous experience of severe side effects from the use of hallucinogens.

14) Use of hallucinogenic substances within the last six months.

15) Blood donation 3 months before and after trial participation.

16) Hemoglobin below 7.8 mmol/L for women and 8.4 mmol/L for men.

17) Ferritin outside the normal range (12–300 µg/L).

18) Body weight below 50 kg.

MRI acquisition and processing

MRI data were acquired on a Siemens Magnetom Prisma 3T scanner using a 32-channel head coil (Siemens AG, Erlangen, Germany). Structural T1- and T2-weighted images were acquired: T1 protocol: Isotropic 0.8 mm resolution, repetition time = 1810 ms, echo time = 2.41 ms, inversion time = 920 ms, and flip angle = 9°. T2 protocol: Isotropic 0.9 mm resolution, repetition time = 3200 ms, echo time = 408 ms). MRI preprocessing and grey matter multispectral segmentation were performed using SPM12 (Functional Imaging Laboratory, the Wellcome Trust Centre for NeuroImaging, London, UK), as previously described ^1^.

PET data acquisition and preprocessing

A full description of the protocols for [^11^C]UCB-J radiosynthesis, PET acquisition and preprocessing has been published previously ^1^. Briefly, participants were scanned with a high-resolution research tomograph (HRRT) PET scanner (CTI/Siemens, Knoxville, TN, USA) for 120 min, starting at the time of the intravenous [^11^C]UCB-J bolus injection. PET data were acquired in 3D list-mode and reconstructed into 40 frames using 3D OP-OSEM algorithm with all standard corrections ^2,3^. Images consisted of 207 planes of 256 x 256 voxels of 1.22x1.22x1.22 mm^3^.

Arterial blood samples were collected during the PET scan to measure total whole blood and plasma radioactivity, unmetabolized radioligand (parent fraction) and radiolabeled metabolites in plasma, in order to establish an arterial input function (AIF), as well as estimate the plasma free fraction of [^11^C]UCB-J (Johansen et al., 2023).

Following motion-correction with the AIR software (Automated Image Registration, v. 5.2.5, LONI, UCLA), individuals’ T1-weighted MR image was co-registered to participants’ PET image using SPM12. Regions of interest (ROIs) were automatically defined using a the PVElab software (https://nru.dk/index.php/allcategories/category/30-software), as previously described ^4^. Mean radioactivity was extracted from 4D PET images for all grey-matter masked ROIs to extract regional time-activity curves.

# Supplementary Results

Figure S1. Latent variable models for the effect of session environment on positive persisting effects (A) and negative persisting effects (B).

Figure S2. Changes in [^11^C]UCB-J BP_ND_ in the frontal cortex (A) and hippocampus (B).

Figure S3. Distribution of MEQ subscale scores for all 15 participants. Columns: median, error bars: IQR.

Figure S4. Distribution of ASC subscale scores for all 15 participants. Columns: median, error bars: IQR.

Figure 5. Pearson and Spearman correlations for individual subfacets of the MEQ, ASC and PEQ questionnaires.

1. Johansen A, Armand S, Plavén-Sigray P, et al. Effects of escitalopram on synaptic density in the healthy human brain: a randomized controlled trial. *Mol Psychiatry*. 2023;28(10):4272-4279. doi:10.1038/s41380-023-02285-8

2. Sureau FC, Reader AJ, Comtat C, et al. Impact of Image-Space Resolution Modeling for Studies with the High-Resolution Research Tomograph. *J Nucl Med*. 2008;49(6):1000-1008. doi:10.2967/jnumed.107.045351

3. Keller SH, Svarer C, Sibomana M. Attenuation Correction for the HRRT PET-Scanner Using Transmission Scatter Correction and Total Variation Regularization. *IEEE T Med Imaging*. 2013;32(9):1611-1621. doi:10.1109/tmi.2013.2261313

4. Svarer C, Madsen K, Hasselbalch SG, et al. MR-based automatic delineation of volumes of interest in human brain PET images using probability maps. *Neuroimage*. 2005;24(4):969-979. doi:10.1016/j.neuroimage.2004.10.017
